# Time Course of Changes in Simulated Keratometry and Total Corneal Refractive Power after Corneal Collagen Cross-Linking for Progressive Keratoconus

**DOI:** 10.1155/2018/2620784

**Published:** 2018-08-12

**Authors:** Masahide Takahashi, Kazutaka Kamiya, Yusuke Kono, Nobuyuki Shoji

**Affiliations:** ^1^Department of Ophthalmology, Kitasato University, Japan; ^2^School of Allied Health Sciences, Kitasato University, Japan

## Abstract

**Purpose:**

To assess the simulated keratometry (Sim K) and the total corneal refractive power (TCRP) in eyes undergoing conventional corneal cross-linking (CXL).

**Methods:**

This study comprised 20 eyes of 20 keratoconic patients (14 men and 6 women; median age (25th and 75th percentile), 26.5 (21.8, 38.0) years) who underwent CXL. The Sim K and TCRP were measured with a rotating Scheimpflug system (Pentacam HR, Oculus), preoperatively and 1, 3, 6, and 12 months postoperatively.

**Results:**

The values of Sim K were 52.65 (46.00, 55.70), 52.45 (45.85, 56.88), 51.70 (45.78, 55.83), 51.40 (45.68, 56.80), and 51.25 (46.08, 56.15) D preoperatively and 1, 3, 6, and 12 months postoperatively, respectively. The corresponding figures of TCRP were 52.10 (45.48, 55.08), 51.30 (45.18, 55.20), 50.95 (45.15, 54.50), 50.00 (45.18, 55.08), and 49.80 (45.48, 54.15) D, respectively. The variances of the Sim K and TCRP data were not statistically significant (p=0.994 and p=0.970, respectively, Kruskal–Wallis test). The Sim K was significantly larger than the TCRP before CXL and at 1, 3, 6, and 12 months after CXL (p<0.001, Wilcoxon signed-rank test).

**Conclusions:**

Not only the Sim K but also TCRP was decreased by approximately 1 D after CXL. The Sim K readings may overestimate the TCRP, even after CXL for progressive keratoconus.

## 1. Introduction

Keratoconus is a progressive noninflammatory disorder characterized by anterior protrusion and thinning of the cornea, deteriorating visual performance with time. The corneal cross-linking (CXL) by means of riboflavin and ultraviolet light has been well established as a therapeutic approach to halt the progression of the disease in eyes with keratoconus [1, 2]. However, we usually evaluated the progression of the disease mainly in terms of the keratometric readings obtained by using a corneal topographer or a autokeratometer, both of which were routinely used in daily practice. These keratometric readings are theoretically calculated based on the assumption that the ratio of the anterior and posterior curvatures remained constant. Moreover, the CXL treatment itself may induce a change in the anterior and posterior corneal curvatures and subsequently alter the actual total corneal power for keratoconus. Hence, these simulated keratometric readings (Sim K) may overestimate the actual total corneal refractive power (TCRP), in not only pre- but also post-CXL treated eyes. However, to the best of our knowledge, the time course of changes in the true corneal power has not so far been extensively investigated in eyes having CXL treatment. It may give us intrinsic insights into the precise changes in the true corneal power, which are essential to determine the precise intraocular lens (IOL) power and/or rigid gas permeable (RGP) lens power in such patients in daily practice. The purpose of the current study is to retrospectively assess the time course of changes in the Sim K and TCRP, in a cohort of progressive keratoconic subjects who underwent conventional CXL treatment.

## 2. Materials and Methods

### 2.1. Study Population

The study protocol was registered with the University Hospital Medical Information Network Clinical Trial Registry (000030659). This retrospective study comprised 20 eyes of 20 keratoconic patients (14 men and 6 women; median age (25th and 75th percentile), 26.5 (21.8, 38.0) years) who underwent standard CXL treatment for progressive keratoconus, and who completed a 1-year follow-up, with good quality scans of corneal tomography measured with a rotating Scheimpflug imaging instrument (Pentacam HR™, Oculus, Wetzlar, Germany). Diagnosis of keratoconus was conducted by one experienced clinician (K.K.) with evident findings characteristic of keratoconus (e.g., corneal topography with asymmetric bow-tie pattern with or without skewed axes) and at least one keratoconus sign (e.g., stromal thinning, conical protrusion of the cornea at the apex, Fleischer ring, Vogt striae, or anterior stromal scar) on slit-lamp examination [3]. Progression was defined as an increase in the maximum keratometric reading of at least 1 diopter (D), or a worsening of corrected visual acuity with an increase of astigmatism ≥1 D confirmed in at least 2 examinations during the preceding 6 to 12 months before treatment. We did not perform CXL in eyes with thinner corneas (the thinnest point < 400 mm), in consideration of the safety issues of corneal endothelial cell density. Eyes with pellucid marginal degeneration, other corneal diseases, and previous ocular trauma or surgery were excluded from the study. The patients were recruited in a continuous cohort. The patients who wore rigid gas permeable and soft contact lenses were asked to stop wearing them for 3 and 2 weeks before this evaluation, respectively, in order to exclude the effect of wearing contact lenses [4, 5]. We randomly enrolled only one eye per subject for statistical analysis. The sample size in the present study offered 80.7% statistical power at the 5% level in order to detect a 1-D difference in the corneal refractive power, when the SD of the mean difference was 1.5 D. This retrospective review of the data was approved by the Institutional Review Board at Kitasato University and followed the tenets of the Declaration of Helsinki. Our Institutional Review Board waived the requirement for informed consent for this retrospective study.

### 2.2. Corneal Cross-Linking

The standard CXL technique was applied in accordance with the Dresden protocol [1]. In brief, after topical anesthesia, we removed the corneal epithelium from a central circular area of 8 mm in diameter using a blunt spatula. Then, we topically administrated riboflavin 0.1% solution every 2 minutes for 30 minutes and confirmed that adequate riboflavin was penetrated to the anterior chamber using a slit-lamp microscopy with a blue filter. We used an Opto XLink Corneal Cross-Linking System™ (North Miami, FL, US), in order to deliver UVA irradiation at the wavelength of 370 nm and a surface irradiance of 3 mW/cm^2^ for 30 minutes. During the irradiation, we applied riboflavin solution every 5 minutes. After treatment, we used topical steroidal and antibiotic medications 4 times daily for 2 weeks, with the dose being reduced gradually thereafter, with an extended-wear bandage contact lens until reepithelialization.

### 2.3. Assessment of Simulated Keratometry and Total Corneal Refractive Power

The values of Sim K and TCRP on the central 15° ring (equal to the 3.0-mm ring) around the corneal apex were automatically measured with the Scheimpflug imaging system (Pentacam HR, software version 1.20), preoperatively and 1, 3, 6, and 12 months postoperatively, as described previously [4, 5]. We checked image quality for each eye and selected only one examination with a high quality factor. Sim K is determined as the average keratometry, calculated by using the standard keratometric index (1.3375) and the radius of anterior corneal curvature. TCRP is determined as the total refractive power, calculated by ray tracing through the anterior and posterior corneal surfaces according to Snell's law.

### 2.4. Statistical Analysis

We conducted statistical analyses by using a commercially available statistical software (Bell Curve for Excel, Social Survey Research Information Co, Ltd., Tokyo, Japan). The normality of all data samples was first checked by the Kolmogorov–Smirnov test. Since all data did not fulfill the criteria for normal distribution, the Wilcoxon signed-rank test was used to compare the pre- and post-CXL treatment. The Kruskal–Wallis test was used to assess the time course of changes in Sim K and TCRP, with the Steel–Dwass test being employed for multiple comparison. Unless otherwise indicated, the results are expressed as the median (25th and 75th percentile), and a value of p<0.05 was considered statistically significant.

## 3. Results

### 3.1. Patient Demographics

The demographics of the study population was summarized in Table 1. We found no intraoperative complications in this series.

### 3.2. Visual and Refractive Outcomes

**Figure 1:**
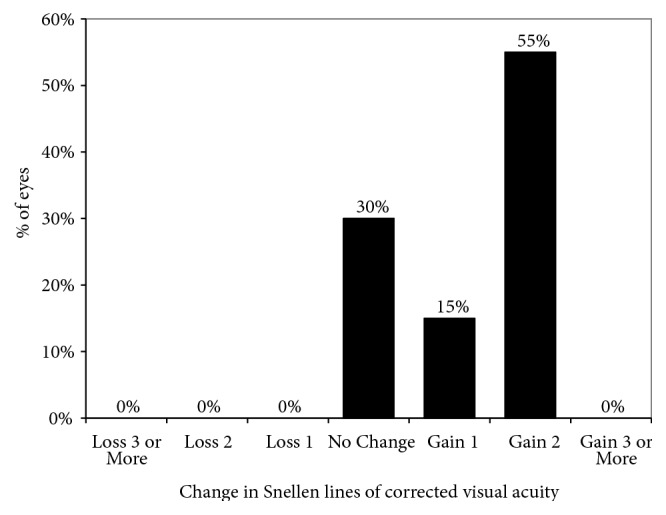
Changes in corrected visual acuity 1 year after corneal cross-linking (CXL).

Uncorrected visual acuity was not significantly changed, from 1.00 (0.70, 1.40) logMAR preoperatively to 1.00 (0.52, 1.27) logMAR postoperatively (Wilcoxon signed-rank test, p=0.173). Corrected visual acuity was significantly improved, from 0.40 (0.10, 0.52) logMAR preoperatively to 0.10 (0.00, 0.22) logMAR postoperatively (p=0.008). Six eyes (30%) showed no change in corrected visual acuity, 3 eyes (15%) gained 1 line, and 11 eyes (55%) gained 2 lines, 1 year postoperatively (Figure 1). Manifest spherical equivalent was not significantly changed, from -2.31 (-6.00, -0.69) D preoperatively to -1.75 (-4.06, -0.22) D logMAR postoperatively (p=0.617). Manifest astigmatism was not significantly changed, from 3.00 (1.25, 5.00) D preoperatively to 2.25 (0.00, 5.00) D postoperatively (p=0.423).

### 3.3. Simulated Keratometry and Total Corneal Refractive Power

**Figure 2:**
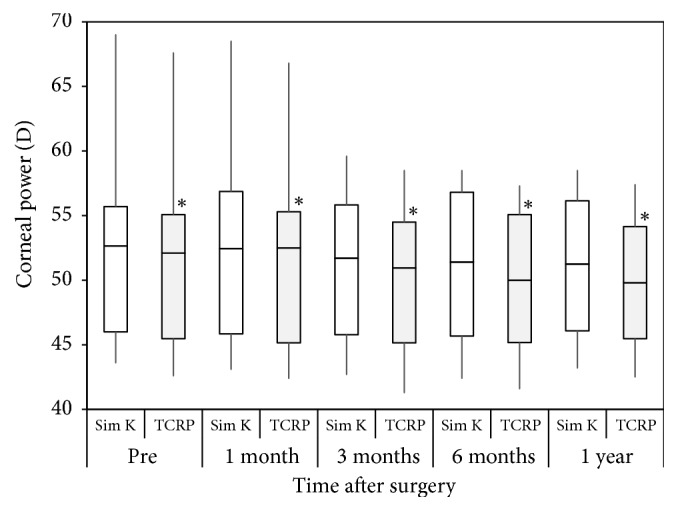
Time courses of the simulated keratometry (Sim K) and total corneal refractive power (TCRP) after corneal cross-linking (CXL). The Sim K was significantly larger than the TCRP before CXL and at 1, 3, 6, and 12 months after CXL. The results are expressed as median ± quartiles. *∗*p<0.001.

The time courses of the Sim K and TCRP are shown in Figure 2. The variance of the Sim K data was not statistically significant (p=0.994, Kruskal–Wallis test). Multiple comparisons demonstrated no significant differences between measurements made before CXL and at 1, 3, 6, and 12 months after CXL (p=1.000, 1.000, 0.998, and 0.999, respectively, Steel–Dwass test). The variance of the TCRP data was not also statistically significant (p=0.970, Kruskal–Wallis test). Multiple comparisons also demonstrated no significant differences between measurements made before CXL and at 1, 3, 6, and 12 months after CXL (p=1.000, 0.994, 0.983, and 0.992, respectively). The Sim K was significantly larger than the TCRP before CXL and at 1, 3, 6, and 12 months after CXL (p<0.001, Wilcoxon signed-rank test). This difference between the Sim K and TCRP tended to be larger with time.

### 3.4. Corneal Thickness

**Figure 3:**
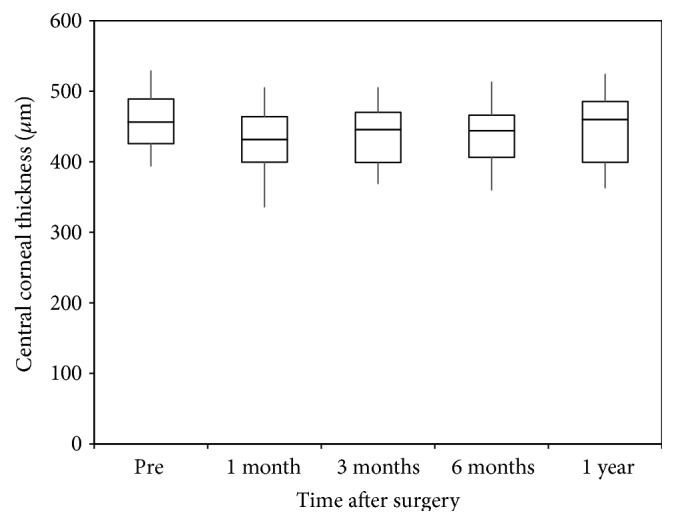
Time course of central corneal thickness after corneal cross-linking (CXL). Multiple comparisons demonstrated no significant differences between measurements made before CXL and at 1, 3, 6, and 12 months after CXL. The results are expressed as median ± quartiles.

Time course of changes in central corneal thickness is shown in Figure 3. The variance of the central corneal thickness data was not statistically significant (p=0.194, Kruskal–Wallis test). Multiple comparisons demonstrated no significant differences between measurements made before CXL and at 1, 3, 6, and 12 months after CXL (p=0.108, 0.993, 0.781, and 0.949, respectively, Steel–Dwass test).

### 3.5. Endothelial Cell Density

Endothelial cell density was not significantly changed, from 2738 (2491, 2906) cells/mm^2^ preoperatively to 2651 (2425, 2887) cells/mm^2^ postoperatively (p=0.140).

### 3.6. Adverse Events/Complications

No eyes showed any progression of the disease at any time after CXL. Three eyes (15%) showed a transient mild haze formation at 1 to 3 months after CXL. Otherwise, we found no vision-threatening complications such as severe haze formation, severe corneal endothelial cell loss (≥10%), or infection.

## 4. Discussion

In the present study, our findings showed that there were no significant changes in Sim K and TCRP before and after CXL treatment, but that both Sim K and TCRP were decreased by approximately 1 D one year after CXL treatment. Vinciguerra et al. reported that the average keratometry showed a mean reduction of 1.06 D 2 years after CXL [6]. Doors et al. described that no significant keratometric changes were observed at 3, 6, and 12 months postoperatively compared with preoperatively, after an initial steepening of maximal keratometry values [7]. Lamy et al. found a mean reduction in Sim K of 0.41, 0.41, 0.33, and 0.61 D after 3 months, 6 months, 1 year, and 2 years of CXL treatment, respectively [8]. The current findings of a reduction in the mean keratometry were comparable with these previous findings [6–8]. Although uncorrected visual acuity was not significantly changed, corrected visual acuity was significantly improved after CXL treatment. The CXL treatment may improve the irregular shape of the cornea, resulting in a decrease in higher-order aberrations and a subsequent improvement in correct visual acuity in the study population. As far as we can ascertain, this is the first study to assess the time course of the actual corneal power in eyes undergoing CXL for progressive keratoconus. Our findings also showed that the Sim K was significantly larger than the TCRP not only preoperatively, but also postoperatively, and that the difference between the Sim K and TCRP tended to be larger with time. It is suggested that the Sim K readings may overestimate the TCRP even after CXL for progressive keratoconus, especially when longer time has passed after CXL.

It is still challenging to accurately determine IOL power for post-CXL treated eyes in daily practice. We assume that it is clinically helpful for understanding the precise change in corneal power after CXL for keratoconus. The overestimation of the corneal refractive power may lead to the selection of the lower IOL power, resulting in a hyperopic refractive error in eyes with keratoconus. Actually, Leccisotti et al. stated that the IOL exchange due to imprecise IOL power occurred in 32% after refractive lens exchange in keratoconic patients [9]. Watson et al. stated that the use of actual keratometric readings can result in a large hyperopic error for severe keratoconus [10]. Park et al. reported that a hyperopic shift was noted since localized corneal posterior elevation is not reflected in conventional IOL power calculation for posterior keratoconus [11]. Camps et al. reported that the use of a single value of the keratometric index for the calculation of the total corneal power in keratoconus has been shown to be imprecise, leading to inaccuracies in the detection and classification of this corneal condition [12]. Furthermore, the CXL treatment may alter both anterior and posterior corneal curvatures and thus result in the actual total corneal power in keratoconic eyes. We should be aware that there is a need for optimizing IOL power when we calculated IOL power using the conventional keratometric readings, not only before CXL but also after CXL.

This study has at least two limitations. One is that we determined the Sim K and TCRP on the 3.0-mm ring only using the Scheimpflug imaging system, because this measurement is considered to be simple and easy to quantitatively grasp corneal refractive power for keratoconus. However, anterior segment optical coherence tomographer may have advantages over the Scheimpflug camera in terms of the accuracy as well as the reproducibility, especially in keratoconic eyes with corneal opacity [13]. Another limitation is that we did not evaluate the repeatability of the corneal power measurements in this cohort. However, we previously confirmed the good repeatability of the Sim K and TCRP measurements for keratoconus [5]. Additionally, the Scheimpflug system has been shown to have an excellent repeatability of the corneal curvature measurements even in keratoconic eyes [14, 15]. Hence, we believe that the instrument offers clinically reasonable repeatability even in the assessment of corneal refractive power in post-CXL keratoconic eyes.

In conclusion, our findings indicate that CXL was effective in halting the progression of the disease in eyes with keratoconus, and both Sim K and TCRP were decreased by approximately 1 D after CXL. The Sim K readings may overestimate the TCRP, even after CXL for progressive keratoconus. We believe that this information was simple, but helpful for understanding the actual corneal power in post-CXL treated eyes, especially when we calculate the precise IOL power and/or RGP lens power in such patients.

## Figures and Tables

**Table 1:** Preoperative demographics of the study population in eyes undergoing corneal cross-linking for progressive keratoconus.

Preoperative demographics (median (25th and 75th percentile))	
Number of eyes	20
Male : Female	14 : 6
Age	26.5 (21.8, 38.0) years
Uncorrected visual acuity (logMAR)	1.00 (0.70, 1.40)
Corrected visual acuity (logMAR)	0.40 (0.10, 0.52)
Manifest spherical equivalent	-2.31 (-6.00, -0.69) D
Manifest cylinder	3.00 (1.25, 5.00) D
Sim K	52.65 (46.00, 55.70) D
TCRP	52.10 (45.48, 55.08) D

logMAR: logarithm of the minimal angle of resolution, D: diopter, Sim K: simulated keratometry, TCRP: total corneal refractive power.

## Data Availability

The data used to support the findings of this study are available from the corresponding author upon request.
